# Wnt/*β*-Catenin Pathway Balances Scaffold Degradation and Bone Formation in Tissue-Engineered Laminae

**DOI:** 10.1155/2021/8359582

**Published:** 2021-09-11

**Authors:** Hailong Li, Linli Li, Yiqun He, Wei Mao, Haofei Ni, Aolei Yang, Feizhou Lyu, Youhai Dong

**Affiliations:** ^1^Department of Orthopedics, Shanghai Fifth People's Hospital, Fudan University, China; ^2^Department of Orthopedics, Huashan Hospital, Fudan University, China

## Abstract

Tissue engineering provides a promising way for the regeneration of artificial vertebral laminae. Previous studies have confirmed the feasibility of reconstructing vertebral laminae via hydroxyapatite-collagen I scaffolds and mesenchymal stromal cells. However, there were no studies exploring the degradation of hydroxyapatite-collagen I scaffolds and the function of Wnt/*β*-catenin pathway in the process. In this study, tissue-engineered laminae (TEL) were constructed by nanohydroxyapatite/collagen I scaffolds and umbilical cord Wharton's Jelly mesenchymal stromal cells (WJ-MSCs). Cell attachment was observed by scanning electron microscopy, and cell viability was confirmed by Live/Dead staining. The rat models were randomly divided into control and *β*-catenin inhibition groups. Vertebral lamina defect rat models were made on the fifth lumbar vertebrate, and TEL was implanted into the defect site. After 14 weeks, the newborn laminae were harvested for microcomputed tomography, histology, or transcriptional profile analysis. We found that, for the control group, the newborn lamina formation matched with the scaffold degradation and complete newborn laminae formed at the 14th week; for the *β*-catenin inhibition group, the scaffold degradation rate overrated the lamina formation and no complete artificial laminae were formed at the 14th week. In addition, the osteoclastic genes, such as Cathepsin K or RANKL, in the control groups were significantly lower than the *β*-catenin inhibition group, and the antiosteoclastic gene, OPG, in the control group was significantly higher than the *β*-catenin inhibition group. In conclusion, inhibition of Wnt/*β*-catenin pathway led to speedy scaffold degradation and deferred artificial lamina formation. Wnt/*β*-catenin pathway played a critical role in maintaining the balance between scaffold degradation and bone formation in the process of vertebral lamina reconstruction.

## 1. Introduction

Laminectomy was a routine surgical protocol for spinal diseases with spinal stenosis [1, 2]. Postoperative epidural scar adhesion can lead to persistent back pain and difficulty for reoperation [3–6]. Tissue engineering techniques have been successfully used to reconstruct the epidural fat or vertebral laminae to avoid epidural scar adhesion in animal studies [7–11].

The common tissue engineering approach involves the use of a biocompatible scaffold, cells, and/or a combination of bioactive molecules such as growth factors and cytokines. Due to the particular structures of the spinal canal, soft biomaterials were favored for the reconstruction of vertebral laminae avoiding compression of the spinal cord after implantation [7]. Nanohydroxyapatite/collagen I scaffolds (nHE/COL) have been well characterized and commercialized for bone defect repair [12, 13]. Its excellent osteoinduction and mechanical properties make it an ideal material for the construction of tissue-engineered laminae (TEL) [14, 15]. Previous studies [7, 16, 17] have successfully constructed TEL with nHE/COL scaffolds and mesenchymal stromal cell (MSC) and reconstructed vertebral laminae. However, the relationship between nHE/COL scaffold degradation and lamina formation remains unclear.

Wnt/*β*-catenin signaling pathway played a crucial role in bone regeneration [18]. Transcriptional profiling and spatial gene expression analysis have found a series of Wnt signaling molecules that are involved in the process of fracture healing [19–21]. Besides, Wnts and their antagonists also exhibit a distinct temporal expression pattern by targeting different cell lineages during the bone regeneration process [18]. Specifically, Wnts induce self-renewal and proliferation in skeletal stem/progenitor cells during the early stages of fracture healing, and once osteogenic differentiation has been triggered in these cells, Wnts then activate the differentiation cascade [18]. Moreover, Wnts can induce the expression of OPG on osteoblasts by regulating the differentiation of osteoclasts and affecting the function of osteoclasts, which also ultimately affects the bone resorption process [22]. Thus, Wnt/*β*-catenin signaling pathway is involved in bone remodeling by regulating both bone resorption and bone formation processes.

In this study, we speculated that Wnt/*β*-catenin signaling pathway might regulate the balance between scaffold degradation and bone formation and play an important role in the reconstruction of vertebral laminae. Tissue-engineered laminae (TEL) were constructed using rat umbilical cord Wharton's Jelly-derived MSC and nHE/COL scaffold. The rat models were randomly divided into control and *β*-catenin inhibition groups. Vertebral lamina defect rat models were made, and TEL was implanted into the defect site. After 14 weeks, the newborn laminae were harvested for microcomputed tomography, histology, or transcriptional profile analysis.

## 2. Methods

### 2.1. Ethics Statement

All animal experiments were performed in the Animal Facility of East China Normal University and according to the protocol (Protocol number: 20150482A168) authorized by the Animal Care and Use Committee of Fudan University.

### 2.2. Cell Culture

Rat mesenchymal stromal cells derived from umbilical cord Wharton's Jelly were purchased from Otwo Biotech (Guangzhou, China). All cells were cultured in Dulbecco's modified Eagle's medium (DMEM; Hyclone, UT, USA) supplemented with 10% fetal bovine serum (FBS; Biological Industries, Beit HaEmek, Israel).

### 2.3. Construction of Tissue-Engineered Laminae (TEL)

The nHE/COL scaffold was bought from the Beijing Allgens Medical Science & Technology Co., Ltd. Under sterile condition, the nHE/COL scaffold was cut to the size of 8 mm × 6 mm. Rat MSC at passage 4-7 was trypsinized and resuspended in media at a concentration of 1 × 10^6^/ml. Then, 100 *μ*l cell suspensions were pipetted on one side of each scaffold, and after 30 min, 100 *μ*l cell suspensions were pipetted on the other side. All constructs were placed in culture medium before implantation.

### 2.4. Live/Dead Staining

Cell viability of MSC in TEL was measured with a Live/Dead assay kit (Best-Bio, Shanghai, China). Experiments were performed according to the manufacturer's instructions. The representative images were obtained using a ZEISS confocal fluorescence microscope (ZEISS, Jena, Germany).

### 2.5. Scanning Electron Microscope

The TEL was fixed with electron microscopy fixative solution (Severicebio, Wuhan, China), dehydrated, mounted on an aluminum stub, and sputter-coated with gold-palladium for 30 seconds. The morphology of TEL and MSC adhesion on the scaffolds was then viewed on a scanning electron microscope (Hitachi, Tokyo, Japan).

### 2.6. Animal Studies

The construction of vertebral lamina defect rat models was performed as previously described [17]. Briefly, spinous processes and interspinous ligaments were removed to expose the vertebral laminae, a bone defect measuring 8 mm × 6 mm was created in the vertebral laminae, and then, the TEL was placed and fixed in the bone defect. For the *β*-catenin inhibition group, the rats were injected intraperitoneally with XAV-939 (MCE, NJ, USA) according to the standard of 4 mg/kg at the frequency of twice a week for the first two weeks and once a week. For the control group, the rats were injected intraperitoneally with the same dose of saline.

### 2.7. Micro-CT Examination

The target vertebrates were harvested at the 2nd, 4th, 6th, 10th, and 14th weeks and fixed in 4% (w/v) paraformaldehyde. The specimens were examined using High Resolution in vivo X-ray Microtomograph System (Bruker, Bremen, Germany). The 3D model was reconstructed manually using the NRecon Reconstruction software (1.7.4.2, Bruker) and analyzed using the CTAn software (1.18.8.0, Bruker).

### 2.8. Histological Staining

After the micro-CT examination, the tissue specimens were decalcified with 10% ethylenediaminetetraacetic acid for 4 weeks. Tissue sections with 6 *μ*m thickness were cut on a microtome and mounted onto glass slides. The sections were processed for routine histological analysis by hematoxylin-eosin (HE), Goldner's trichrome, tartrate-resistant acid phosphatase (TRAP), and immunohistochemistry (IHC) staining.

### 2.9. IHC Staining

Immunohistochemistry was performed as previously described [17]. The following antibodies were used: anti-RANKL rabbit polyclonal (GB11235, Severicebio), anti-osteoprotegerin rabbit polyclonal (GB11151, Severicebio), anti-OCN rabbit polyclonal (GB11233, Severicebio), and horseradish peroxidase- (HRP-) conjugated secondary antibodies (GB23303, Severicebio). The percentage of positive staining area was analyzed by the plugins of IHC Profiler in the Image J software (Version: 1.8.0_112, NIH, USA).

### 2.10. Reverse Transcription PCR

Total RNA was isolated from cells or bone tissue using TRIzol Reagent (Invitrogen) according to the manufacturer's instructions. cDNA was synthesized from total RNA (500 ng) using a reverse transcription kit (Takara, Tokyo, Japan). qPCR was performed in triplicate using 1 *μ*L of cDNA in a standard SYBR premix Ex Taq (Takara) on the Applied Biosystems 7500 Real-Time PCR Detection System (Applied Biosystems, CA, USA). GAPDH served as an internal control. The following primers were used: GAPDH, 5′-GGCACAGTCAAGGCTGAGAATG-3′ and 5′-ATGGTGGTGAAGACGCCAGTA-3′; RANKL, 5′-ATGATGGAAGGTTCGTGG-3′ and 5′-GGACAGACTGACTTTATGGG-3′; OPG, 5′-AGACCGTGAAACAGGAGTG-3′ and 5′-ACCTGAGAAGAACCCATCC-3′; and CTSK, 5′-GAAGAAGACTCACCAGAAGCAG-3′ and 5′-TCCAGGTTATGGGCAGAGATT-3′. The relative gene expression was calculated using the following equation: ΔCt = Ct (test genes) − Ct (GAPDH); fold change = 2^(−ΔCt)^.

### 2.11. Statistical Analysis

Statistical analysis was conducted using GraphPad Prism version 6.02 software program for Windows (GraphPad, CA, USA). The Student *t*-test was used for comparison between groups. All tests were two-sided, and *P* values <0.05 were considered to be statistically significant.

## 3. Results

### 3.1. TEL Construction

**Figure 1:**
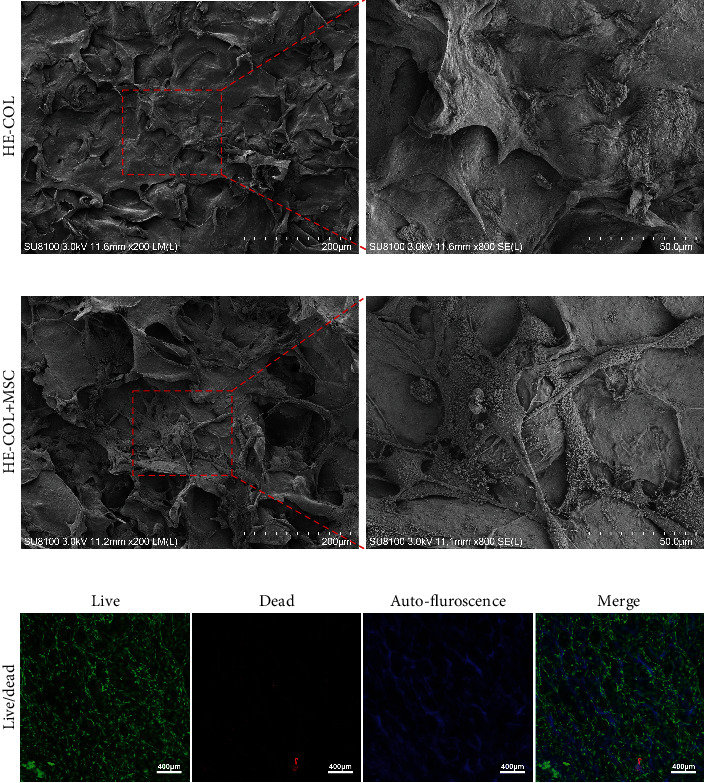
(a) SEM images of nHA/COL scaffolds; (b) SEM images of tissue-engineered laminae (TEL); (c) Live/Dead staining of TEL.

SEM scanning was used to observe the surface morphology of the scaffold. We found that there were enormous micropores and irregular lamellar structures distributed on the surface of nHA/COL scaffolds (Figure 1(a)). MSC formed finger-like filopodia and tightly adhered to the lamellar structure of scaffolds (Figure 1(b)). The Live/Dead staining showed that almost all the MSC survived in the nHA/COL scaffold, and the cells tightly adhered to the lamellar structure of the scaffold (Figure 1(c)). The scaffold has blue autofluorescence (Figure 1(c)).

### 3.2. Micro-CT Examination

**Figure 2:**
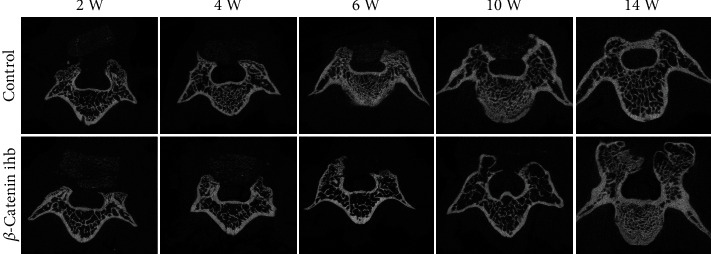
Micro-CT scanning of TEL in the control and *β*-catenin inhibition groups at the 2nd, 4th, 6th, 10th, and 14th weeks.

In the control group, the newborn laminae gradually grew from bilateral vertebral pedicles to the middle and formed complete artificial laminae at the 14th week, and the scaffold degradation and trabecular bone formation proceeded orderly and alternately. In the *β*-catenin inhibition group, the scaffold degradation rate was significantly higher than that of the control group, and bone formation rate was significantly lower than that of the control group. In addition, its scaffold degradation rate was dominant over the bone formation, almost all the scaffold degraded at the 10th week, the newborn laminae grew slowly after the 10th week, and no complete artificial laminae were formed at the 14th week (Figure 2).

### 3.3. Goldner's Trichrome Staining

**Figure 3:**
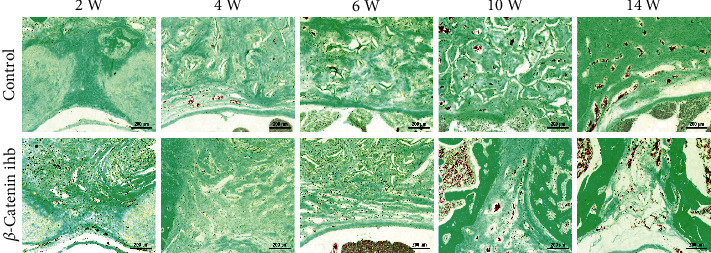
Goldner's trichrome staining of TEL at the 2nd, 4th, 6th, 10th, and 14th weeks.

New bone formation was evaluated by Goldner's trichrome staining. Bone and bone-like tissues are presented as a substructure visualized in green. In the control group, the new trabecular structure formed and bone mineral deposited at the 10th week, and premature bone structure formed at the 14th week. In the *β*-catenin inhibition group, the scaffold degraded completely at the 10th week, the artificial laminae stopped growing, and there was still a lamina defect with the size of 400-600 *μ*m at both the 10th and 14^th^ weeks (Figure 3).

### 3.4. Scaffold Degradation and Bone Formation

**Figure 4:**
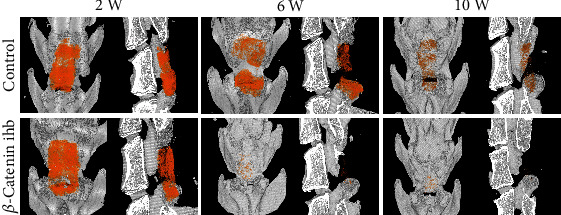
3D reconstruction images at the 2nd, 6th, and 10th weeks; the pseudo orange color indicates the TEL.

**Figure 5:**
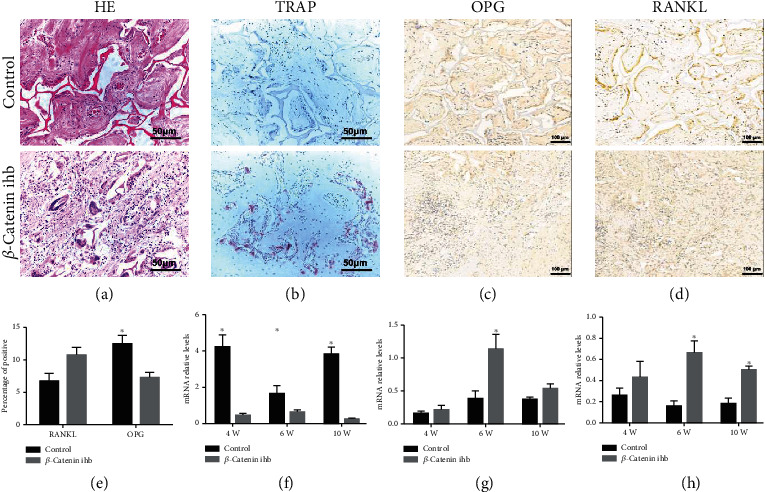
(a) HE staining of the newborn laminae at the 10th week; (b) TRAP staining of the newborn laminae at the 10th week; IHC staining of (c) OPG and (d) RANKL at the 10th week; (e) percentage of positive staining of RANKL and OPG at the 10th week; mRNA expression levels of (f) OPG, (g) RANKL, and (h) CTSK. ^∗^*P* < 0.05 (control vs. *β*-catenin inhibition group at the same time point).

**Figure 6:**
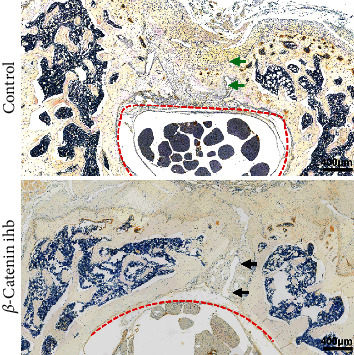
IHC staining of OCN at the 14th week. Green arrow indicates the newborn complete vertebral laminae in the control group, black arrow indicates the lamina defect in the *β*-catenin inhibition group, and red dish line indicates the vertebral canal.

The scaffold degradation rate was analyzed by the 3D reconstruction images. The scaffold degradation rate in the control group was significantly lower than that of the *β*-catenin inhibition group (Figure 4). In the *β*-catenin inhibition group, almost all the scaffold degraded at the 6th and 10th weeks (Figure 5(a)). The HE staining also showed that the scaffold degraded completely at the 10th week in the *β*-catenin inhibition group (Figure 5(b)). TRAP staining showed that the osteoblast numbers in the *β*-catenin inhibition group were significantly higher than that of the control group (Figure 5(c)). The expression levels of OPG in the control group were statistically higher than those in the *β*-catenin inhibition group at the 10th week, while the RANKL expression showed no statistical difference at the 10th week (Figures 5(c)–5(g)) The mRNA expression levels of *CTSK* in the *β*-catenin inhibition group were also statistically higher than those in the control group at both the 6th and 10th weeks (Figure 5(h)) The newborn laminae were also confirmed by IHC staining of OCN at the 14th week (Figure 6).

## 4. Discussion

In this study, we investigated the role of Wnt/*β*-catenin signaling pathway in the balance of scaffold degradation and bone formation during vertebral lamina reconstruction. We found that, with the inhibition of *β*-catenin, the newborn laminae increased the expression levels of osteoclastic markers and the number of osteoclasts, thereby promoting the scaffold degradation and deferring the lamina formation.

Previous studies showed that the OPG-RANK-RANKL system plays the principal role in determining the balance between bone resorption and bone formation [22, 23]. Osteoblastic cell lineages can secrete RANKL, which binds to the RANK receptor on the osteoclast or osteoclast progenitor cells and activates the transcription of osteoclastic genes [22, 23]. Osteoblastic cell lineages can also secrete OPG, which can competitively inhibit the binding between RANKL and RANK, thereby inhibiting the formation and differentiation of osteoclasts [22, 23]. *β*-Catenin can induce the expression of osteoclast inhibitor OPG and regulate osteoclast differentiation, affecting bone resorption ultimately [24]. In this study, we found that the expression levels of OPG in the control group were statistically higher than those in the *β*-catenin inhibition group at all time points, while the expression levels of *RANKL* in the control group were only statistically lower than those in the *β*-catenin inhibition group at 6th week. The regulatory function of Wnt/*β*-catenin signaling pathway in the osteoclast differentiation was mainly played by promoting the expression of OPG.

The coupling balance between scaffold degradation and bone formation is requisite for bone reconstruction [25–27]. The dominance of scaffold degradation inhibits the migration and mineralization of osteoblasts, while the constrained degradation of scaffolds would squeeze the space for osteoblast proliferation [28]. In this study, the scaffold degradation rate in the *β*-catenin inhibition group was dominant over bone formation rate. Almost all the scaffold degraded at the 6^th^ week, and the bone formation retarded thereafter and could not form complete artificial laminae at the 14th week. In the control group, scaffold degradation and bone formation maintained synchronous balance and realized artificial lamina reconstruction at the 14th week. It demonstrated that *β*-catenin inhibition could expedite the degradation of nHE/COL scaffolds and break down the normal balance between scaffold degradation and bone formation, which was detrimental to the reconstruction of artificial vertebral laminae.

Scaffolding biomaterials, such as natural collagen, synthetic polymers, ceramics, inorganic biomaterials, and their hybrid combinations, have been widely used for bone tissue engineering, due to their outstanding biomechanical, osteoconductive, and biodegradable properties [29]. Bone regeneration is closely associated with Wnt/*β*-catenin signaling pathway [18]. Many biomaterials, such as titanium with Ti-Nano, laponite-guanidinylated chitosan hydrogels, intrafibrillar mineralized collagen, hydroxyapatite, and gold nanoparticle-loaded hydroxyapatite composites, possessed great regenerative potential because they can activate Wnt/*β*-catenin signaling pathway and thereby promoting bone regeneration [30–33]. Bone progenitor stem cells, scaffolding biomaterials, and Wnt/*β*-catenin signaling pathway must rely on each other to realize successful bone regeneration [19].

Many studies have clearly elucidated the role of Wnt/*β*-catenin signaling pathway in promoting osteoblast proliferation, migration, and osteogenic differentiation [18, 19]. Previous studies mostly focused on the role of biomaterials in the activation of Wnt/*β*-catenin signaling pathway, osteogenic differentiation, and inhibition of osteoclastogenesis [20, 22, 24]. In this study, we elucidated the role of Wnt/*β*-catenin signaling pathway in inhibiting osteoclast genesis and scaffold degradation and thus in maintaining the balance between scaffold degradation and bone formation. It demonstrated that Wnt/*β*-catenin signaling pathway regulated the reconstruction of tissue-engineered bone in several different ways, which needs more attention in further study.

## 5. Conclusion

In the process of vertebral lamina reconstruction, Wnt/*β*-catenin pathway could decrease the degradation rate of nHA/COL scaffold mainly by decreasing the expression of OPG and promoting osteoclast genesis. Inhibition of *β*-catenin led to speedy scaffold degradation and deferred artificial lamina formation, disturbing the balance between scaffold degradation and bone formation. This study advances our understanding of the role of Wnt/*β*-catenin pathway in bone tissue engineering.

## Data Availability

The datasets used during the current study are available from the corresponding author on reasonable request.
